# Influencing Factors of Medical Narrative Competence Among Clinical Nurses in China: A Systematic Review and Meta‐Analysis

**DOI:** 10.1155/jonm/5040520

**Published:** 2026-11-03

**Authors:** Yuqin Li, Rong Hu, Li Tang, Feng Chen, Wei Yang, Ying Xiao, Huilu Yao, Hongxiu Wang, Qin Mao, Xiaorong Mao

**Affiliations:** ^1^ Department of Cancer Center Sichuan Provincial People’s Hospital School of Medicine University of Electronic Science and Technology of China Chengdu Sichuan China uestc.edu.cn; ^2^ Department of Vascular Surgery Sichuan Provincial People’s Hospital School of Medicine University of Electronic Science and Technology of China Chengdu Sichuan China uestc.edu.cn; ^3^ Department of Thoracic Surgery Sichuan Provincial People’s Hospital School of Medicine University of Electronic Science and Technology of China Chengdu Sichuan China uestc.edu.cn; ^4^ School of Medicine University of Electronic Science and Technology of China Chengdu Sichuan China uestc.edu.cn; ^5^ College of Nursing Chengdu University of Traditional Chinese Medicine Chengdu Sichuan China cdutcm.edu.cn; ^6^ College of Nursing North Sichuan Medical College Nanchong Sichuan China nsmc.edu.cn; ^7^ Department of Outpatient Pediatrics Sichuan Provincial People’s Hospital School of Medicine University of Electronic Science and Technology of China Chengdu Sichuan China uestc.edu.cn; ^8^ Department of Nursing Sichuan Provincial People’s Hospital School of Medicine University of Electronic Science and Technology of China Chengdu Sichuan China uestc.edu.cn

**Keywords:** clinical nurses, influencing factors, meta-analysis, narrative competence, narrative medicine

## Abstract

**Aim:**

To investigate the current level of and influencing factors for medical narrative competence among clinical nurses in China.

**Background:**

In China, clinical nurses generally demonstrate insufficient medical narrative competence. This condition is associated with suboptimal delivery of humanistic nursing services, frequent nurse–patient frictions, and diminished professional identity among nursing staff. It is essential to systematically explore the current situation and relevant influencing factors, so as to enhance clinical nurses’ medical narrative competence and improve the overall quality of nursing services.

**Methods:**

Systematic searches were conducted to retrieve literature on the current status and influencing factors of medical narrative competence among clinical nurses in China from 11 databases, including China National Knowledge Infrastructure (CNKI), Wanfang Database, VIP Database, China Biomedical Literature Database (CBM), PubMed, Embase, Scopus, Cochrane Library, Web of Science, PsycINFO, and the Cumulative Index to Nursing and Allied Health Literature (CINAHL). Meta‐analysis was performed using Stata 15.0 and RevMan 5.4 software.

**Results:**

A total of 34 studies involving 21,455 Chinese clinical nurses were included, and 17 influencing factors were identified. Meta‐analysis showed that the pooled mean total score of medical narrative competence was 147.38 (95% CI: 145.17–149.60). Factors associated with higher medical narrative competence included age > 35 years, working experience ≥ 10 years, senior professional title, head nurse or above position, male nurse status, monthly income ≥ 10,000 CNY, great familiarity with narrative medicine, and previous training experience in medical narrative. By contrast, moderate colleague support and dissatisfaction with nursing work were associated with lower medical narrative competence (all *p* < 0.05).

**Conclusion:**

The medical narrative competence of clinical nurses in China is at the lower end of the moderate level and was associated with multiple individual and occupational factors.

**Implication for Nursing Management:**

These findings may offer preliminary evidence to inform nursing management. Given the factors associated with medical narrative competence, nursing managers may consider developing targeted interventions and training programs to help enhance nurses’ competence and contribute to better nursing care quality.

## 1. Introduction

Narrative medicine (NM), a rigorous intellectual and clinical discipline that leverages narrative skills to deepen scientific understanding of illness, was first proposed by Rita Charon in 2001 [1]. Its core tenet centers on listening to patients’ accounts, interpreting and respecting their experiences, reflecting on their meanings, and acting in patients’ best interests. Over the past 2 decades, NM has gained widespread adoption worldwide [2]. Introduced in China in 2011, it has since been gradually integrated into medical education and clinical practice [3], serving as a key initiative to foster harmonious doctor–patient relationships under the Healthy China 2030 strategy [4].

NM emphasizes integrating narrative thinking into clinical practice and requires medical professionals to possess medical narrative competence. In this study, medical narrative competence is defined as the ability of medical professionals to recognize, absorb, interpret, and respond to patients’ illness experiences, and translate such narrative understanding into appropriate clinical actions [5, 6]. Nurses have both the most frequent patient contact and the most intimate communication with patients in clinical care; consequently, their medical narrative competence may facilitate the implementation of NM. Existing research suggests that nurses’ medical narrative competence may be associated with patient outcomes [7, 8]. Higher medical narrative competence may facilitate nurse–patient communication, contribute to a better patient experience, and be associated with lower levels of nurse burnout. Moreover, this competence may support humanistic nursing care and contribute to better patient health outcomes [9]. Internationally, NM has been applied in clinical care and medical education in countries such as the United States and Italy [10, 11]. The scoping review conducted by Palla et al. [12] summarized relevant research across three dimensions: theory, clinical practice, and education and training, providing an international context for understanding the application of NM.

In contrast, numerous quantitative surveys using standardized measurement tools have been conducted in China in recent years, laying the foundation for further synthesis of the overall level and associated factors of clinical nurses’ medical narrative competence. The Narrative Competence Scale (NCS), developed by Ma et al. [13], is an accessible assessment tool identified in Chinese literature for investigating medical narrative competence. It has undergone empirical validation and been widely adopted in multiple cross‐sectional studies. The scale consists of 27 items covering three dimensions: attentive listening, empathetic responding, and reflective representation. A seven‐point Likert scale is adopted for scoring. The Cronbach’s α coefficient of the overall scale is 0.950, and the content validity index is 0.89, indicating favorable psychometric properties. The total score ranges from 27 to 189. Scores < 145 indicate a weak level, scores of 145–163 represent a moderate level, and scores > 163 correspond to a strong level. Existing studies have shown that Wang et al. [14] reported the NCS scores of nurses in VIP wards as 135.31 ± 16.50, representing a weak level. Ji et al. [15]and Dong et al. [16] reported that the NCS scores of operating room nurses and oncology nurses were 148.68 ± 16.16 and 151.52 ± 14.61, respectively, both indicating a moderate level. These results suggest that medical narrative competence differs across clinical nurse subgroups. Nevertheless, single cross‐sectional surveys cannot capture the overall status of clinical nurses in China, underscoring the need to synthesize the available evidence further.

However, existing studies still have certain limitations. First, most international studies focus on integrating NM into clinical practice and medical education, and few have adopted standardized measurement tools to quantitatively assess the medical narrative competence of practicing clinical nurses. Second, available Chinese studies have explored factors associated with clinical nurses’ medical narrative competence from demographic, occupational, and organizational environmental perspectives. However, the directions of association and statistical significance of factors such as professional title [17, 18], working years [14, 19], and marital status [20, 21] remain inconsistent. Such discrepancies may stem from differences in study populations, variable grouping, sample size, and approaches to adjusting for confounding factors. Therefore, it is necessary to synthesize evidence with clear analytical hierarchies and grouping criteria to evaluate the direction, magnitude, and uncertainty of associations for these relevant factors. Finally, Li et al. [22] conducted a meta‐analysis on a relevant topic and provided preliminary evidence for this field. However, their review failed to synthesize the overall scores of medical narrative competence and lacked subgroup analyses stratified by survey time, clinical department, and geographical region. In addition, several factors, such as gender and employment type, were synthesized from only two studies, resulting in insufficient representativeness of the sample. Furthermore, this previous meta‐analysis lacked methodological quality controls, including PROSPERO registration and trim‐and‐fill correction, indicating considerable room for further improvement in the evidence.

Accordingly, this study aims to synthesize the overall scores of medical narrative competence and conduct subgroup analyses stratified by survey period, clinical department, and geographical region. It will also quantitatively pool the results of univariate analyses with comparable grouped data. This work is expected to provide quantitative references for evaluating the overall level of medical narrative competence among clinical nurses in China, explore differences in scores across subgroups, offer new insights into potential sources of between‐study heterogeneity, and support nursing managers in identifying vulnerable areas and developing training strategies. In addition, the identified associations may inform the assessment of training needs and highlight factors for further investigation in longitudinal and intervention studies.

## 2. Methods

This study was conducted in accordance with the PRISMA statement [23]. The review was registered in the International Prospective Register of Systematic Reviews (PROSPERO) under registration number CRD420251249028.

### 2.1. Search Strategy

A systematic search was conducted in CNKI, Wanfang, VIP, Chinese Biomedical Literature Database (CBM), PubMed, Embase, Scopus, Cochrane Library, Web of Science, PsycINFO, and CINAHL from database inception to 30 August 2026. A combination of subject terms and free‐text keywords was used to identify relevant studies. The search strategy was structured as follows: (“nurses” OR “nursing staff” OR “clinical nurses” OR “registered nurses” OR “on‐the‐job nurses” OR “clinical nursing workers” OR “nursing practitioners”) AND (“medical narrative competence” OR “clinical narrative competence” OR “narrative competence” OR “medical narrative literacy” OR “narrative literacy” OR “narrative competency” OR “narrative medicine” OR “medical narrative” OR “narrative nursing” OR “narrative care” OR “narrative writing” OR “medical narrative writing” OR “clinical narrative” OR “medical narrative competency” OR “clinical narrative competency”) AND (“influencing factors” OR “related factors” OR “associated factors” OR “potential factors” OR “predictors” OR “determinants” OR “independent factors” OR “risk factors” OR “correlation” OR “association”). Search strategies were adapted to the specific syntax requirements of each database. The complete database‐specific search strategies and retrieval counts are provided in Supporting Appendix 1, Section S1. In addition, supplementary searches included screening reference lists and conducting forward citation tracking of included studies, as well as searching gray literature sources (e.g., conference abstracts and dissertations).

### 2.2. Eligibility Criteria

The inclusion criteria of this study were formulated in accordance with the PEOS framework, as detailed in Table 1.

**TABLE 1:** Study eligibility criteria.

Inclusion criteria	Exclusion criteria
1. Population (P): Practicing clinical nurses	1. Only dimension scores reported, or incomplete data that cannot be extracted
2. Exposure (E): Demographic, occupational, organizational, and environmental factors potentially associated with clinical nurses’ medical narrative competence	2. Reviews or protocols
3. Outcome (O): Medical narrative competence assessed using the NCS score, with required statistical data available	3. Duplicate publications
4. Study design (S): Cross‐sectional study	4. Full text unavailable
5. Setting: Clinical nursing settings in Chinese medical institutions, with no restrictions on hospital grade or clinical department	—
6. Other: Full text accessible; independent study data; AHRQ score ≥ 4; published in Chinese or English	—

### 2.3. Data Extraction

All retrieved records were imported into EndNote X9 for deduplication. Two independent reviewers (Mao Q and Tang L) conducted the screening process: they first screened the titles and abstracts of potentially relevant records, followed by full‐text assessment of studies potentially eligible for inclusion. Disagreements were resolved through discussion or, if necessary, by consultation with a third independent reviewer (Xiao Y). Two other reviewers (Yao HL and Wang HX) independently extracted data using a standardized, pilot‐tested data extraction form. The following data were extracted from each included study: (1) The extracted information included the first author, publication year, survey period, province or city, hospital level (tertiary or secondary), sample size, clinical department, influencing factors examined, and study quality score; (2) For the analysis of the overall level, we extracted the sample size and mean ± standard deviation of the total NCS score from each primary study. For the analysis of associated factors, we extracted the names of the influencing factors, grouping schemes, sample size for each group, and the mean ± standard deviation of the total NCS score. This study quantitatively pooled only extractable continuous outcome data from univariate group comparisons reported in the primary studies.

### 2.4. Quality Assessment

Two researchers (Yang W and Chen F) trained in evidence‐based nursing performed quality appraisal of all included studies using the observational study quality assessment tool recommended by the Agency for Healthcare Research and Quality (AHRQ) [24]. Studies with uncertain quality ratings were reevaluated by a third researcher (Hu R). One point was awarded for each item with the response “yes”; zero points were assigned for responses of “no” or “unclear”, yielding a maximum total score of 11. Studies scoring 0–3 were classified as low quality, those scoring 4–7 as moderate quality, and those scoring ≥ 8 as high quality.

### 2.5. Statistical Analysis

For the overall level and subgroup analyses of clinical nurses’ medical narrative competence, the pooled mean of total NCS scores was calculated, along with its 95% confidence interval. For the analyses of associated influencing factors, since all included studies adopted the same assessment tool, the pooled mean differences (MDs) and their 95% confidence intervals were reported. To enhance data stability and the reliability of results, meta‐analyses were conducted only for influencing factors reported in at least five eligible studies. The *I* ^2^ statistic was used to quantify between‐study heterogeneity as a percentage [25]. A fixed‐effects model was adopted when *p* ≥ 0.1 and *I* ^2^ ≤ 50%, indicating nonsignificant heterogeneity across studies. In contrast, a random‐effects model was used when *p* < 0.1 and *I* ^2^ > 50%, reflecting substantial between‐study heterogeneity [26]. Subgroup analyses were performed stratified by the survey start year, geographic region (southern vs. northern China), and clinical department, using a random‐effects model. Sensitivity analyses were performed to assess the robustness of the pooled estimates using two approaches: leave‐one‐out analysis and comparison of fixed‐effects and random‐effects models. Publication bias was assessed using funnel plots, Begg’s test, and Egger’s test when at least 10 studies were included in the meta‐analysis [27].

## 3. Results

### 3.1. Search Results

Database searches retrieved 3079 records from 11 databases. After removing 346 duplicates, 2,733 records were screened by title and abstract, of which 2652 were excluded. The remaining 81 records underwent full‐text review, and 47 were excluded for failing to meet the inclusion criteria. Finally, 34 studies were included in this meta‐analysis. The study selection process is detailed in the PRISMA flow diagram (Figure 1).

**FIGURE 1:**
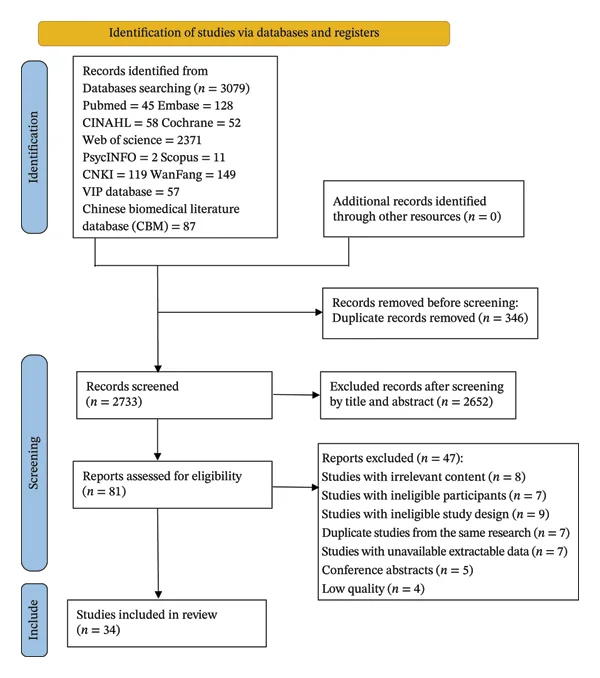
Flowchart of the literature search and selection.

### 3.2. Characteristics of the Included Studies

Of the 34 included studies, 6 were published in English and 28 in Chinese. All studies assessed clinical nurses’ medical narrative competence as the primary outcome using the NCS. The total sample comprised 21,455 nurses, with individual study sample sizes ranging from 94 to 1831. Among studies reporting survey dates, data collection took place between 2019 and 2025 across 18 provinces and municipalities in China. Most studies (*n* = 20) were conducted in southern China, and 13 in northern China. In terms of hospital grade, 23 studies were conducted exclusively in Grade III Level‐A hospitals. Five multicenter studies included general hospitals of multiple grades. One study was conducted in a level‐III hospital without specifying its detailed grade, another in a community hospital, and another in a military hospital. An additional three studies did not explicitly report the grade of the included hospitals. The studies included nurses from multiple departments and clinical settings, including oncology, pediatrics, VIP units, psychiatry, operating rooms, reproductive medicine, community health care, and the Infection Department (Table 2).

**TABLE 2:** Characteristics of included studies.

Author (year)	Province	Grade of medical institution	Survey period	Sample size	Population	Associated factors
Zeng et al., 2024 [28]	Shanxi	Military general hospital	Sep‐Oct 2023	666	Military nurses	2, 7, 16, 19
Dong et al., 2025 [16]	Yunnan	Grade III Level‐A	Jun–Aug 2023	931	Oncology nurses	2, 3, 4, 5, 6, 7, 8, 9, 10, 11, 15, 17, 18, 20, 21
Gao, 2024 [29]	Guangdong	Grade I Level‐A	Mar 2020–Apr 2021	107	Community nurses	2, 3, 4, 7, 9, 10, 11, 12, 15
Gong et al., 2022 [30]	Hunan	Grade III hospital Grade II hospital	Nov 2021–Jan 2022	1629	Psychiatric nurses	19, 22
Guo et al., 2022 [31]	Chongqing	Grade III hospital Grade II hospital	Mar 2022	1519	Clinical nurses	1, 2, 3, 4, 7, 11, 12, 17, 22
Han et al., 2024 [32]	Beijing	Grade III Level‐A	Dec 2023–Jan 2024	319	Pediatric nurses	2, 3, 4, 5, 6, 7, 10, 11, 12, 13, 14, 15, 17, 19, 20, 21, 22, 23
He et al., 2023 [20]	Hunan	Grade III Level‐A	Jan–Feb 2022	1018	Traditional Chinese Medicine nurse	2, 3, 7, 12, 16, 19
Hou et al., 2021 [33]	Guangdong	Grade III Level‐A	May–Jul 2020	292	Clinical nurses	2, 7, 10
Hu et al., 2024 [21]	Shanghai	Grade III Level‐A	Dec 2022	191	Oncology nurses	2, 4, 6, 7, 11, 13, 15
Ji et al., 2025 [15]	Shanxi	Grade III Level‐A	Jan–May 2024	248	Operating room nurses	2, 3, 4, 7, 11, 18
Jia et al., 2024 [34]	Hebei	Grade III Level‐A	Mar–Jun 2023	606	Clinical nurses	2, 3, 4, 12, 13, 15, 24
Li et al., 2024a [35]	Shanxi	Grade III hospital Grade II hospital Grade I hospital	May–Jun 2023	1511	Clinical nurses	2, 3, 4, 5, 7, 8, 11, 15, 17, 18, 19, 21, 23, 24, 25
Liang et al., 2022 [36]	Jiangxi	Grade III Level‐A	Dec 2021–Jan 2022	422	Resident training nurses	4, 15, 19, 23, 24
Shi et al., 2021 [37]	Shandong	Grade III Level‐A	Jul–Dec 2019	352	Oncology nurses	2, 4, 11, 16, 18, 19
Su et al., 2023 [38]	Shandong	Grade III Level‐A	Apr–Jul 2022	214	Clinical nurses	2, 3, 4, 5, 7, 9, 10, 11, 12, 13, 15
Luo, 2024 [39]	Sichuan	Grade III Level‐A	Mar–Aug 2023	608	Clinical nurses	2, 3, 4, 7, 10, 11, 12, 13, 14, 15, 17, 18, 19, 21, 24, 25
Su, 2021 [40]	Jilin	Grade III Level‐A	Dec 2019–Oct 2020	450	Clinical nurses	2, 3, 4, 10, 14, 15, 20, 25
Tian et al., 2021a [19]	Tianjin	Grade III Level‐A	NR	220	Oncology nurses	2, 3, 4, 6, 7, 11, 12, 19
Tian et al., 2021b [41]	Tianjin	Grade III Level‐A	Aug–Oct 2020	350	Clinical nurses	2, 3, 4, 8, 12, 15, 21
Yue et al., 2023 [42]	Sichuan	Grade III Level‐A	Jul 2021–Jan 2022	267	Clinical nurses	2, 3, 4, 7, 11, 12, 15, 18
Zheng et al., 2021 [43]	Guizhou	Grade III Level‐A	Jun 2020	1338	Clinical nurses	2, 3, 4, 5, 7, 11, 12, 13, 15, 18
Mo et al., 2024 [17]	Zhejiang	Grade III hospital Grade II hospital Grade I hospital	NR	122	Reproductive nurses	1, 3, 5
Sun et al., 2025 [18]	Jiangsu	Grade III Level‐A	Jan 2024	115	Pediatric nurses	1, 5, 6, 7, 8
Wang et al., 2024 [14]	Guangdong	Grade III Level‐A	Jun 2022	94	VIP ward nurses	2, 3, 4, 5, 7, 12, 13
Yang et al., 2025 [44]	Hunan	Grade III hospital Grade II hospital	Aug 2022	1792	Clinical nurses	1, 5, 7, 13, 19
Li et al., 2024b [45]	NR	NR	Jan–Feb 2024	1831	Clinical nurses	2, 4, 7, 11, 12, 14, 18, 19
Yu et al., 2024 [46]	Jiangsu	Grade III Level‐A	Dec 2022–Feb 2023	741	Clinical nurses	3, 4, 5, 7, 11, 17, 19
Li, 2023 [47]	Guangdong	NR	Oct–Dec 2021	580	Clinical nurses	2, 3, 4, 7, 8, 10, 11, 17, 21
Li et al., 2022 [48]	Shandong	Grade III Level‐A	Jul–Aug 2021	215	Clinical nurses	2, 3, 6, 10, 11, 15
Chen et al., 2026 [49]	Neimenggu	Grade III Level‐A	Oct 2024–Mar 2025	150	Clinical nurses	2, 3, 4, 7, 11, 12, 13, 15, 17, 19, 20
Xu et al., 2023 [50]	Zhejiang	Grade III Level‐A	Feb 2022	530	Clinical nurses	2, 3, 4, 7, 11, 15
Li et al., 2024c [51]	Liaoning	Grade III Level‐A	Jul 2022	1036	Clinical nurses	5, 7, 10, 13, 15
Dai et al., 2023 [52]	Yunnan	NR	Mar–May 2022	216	Infection control nurse	1, 2, 3, 5, 7, 11, 13, 15, 19
Xie et al., 2021 [53]	Guangdong	Grade III hospital	Oct 2020–Jan 2021	775	Oncology nurses	2, 3, 4, 15

*Note:* (1) Grade of medical institution; (2) Working years; (3) Professional title; (4) Degree; (5) Post; (6) Status as an only child; (7) Marital status; (8) Family background; (9) Family concern; (10) Colleague concern; (11) Gender; (12) Age; (13) Fertility status; (14) Department; (15) Familiar with narrative medicine; (16) Narrative Medicine Knowledge Reserve; (17) Employment method; (18) Income (RMB/month); (19) Narrative Nursing Training (Knowledge) Experience; (20) Personality Traits; (21) Nursing Job Satisfaction; (22) Willingness to Promote Narrative Nursing; (23) Narrative Medicine‐Related Practical Experience; (24) Attitude Toward the Implementation of Narrative Nursing; (25) Professional Identity and Achievement in the Nursing Profession.

Abbreviation: NR, not reported.

Quality appraisal scores of the included studies ranged from 4 to 9, with a mean (SD) of 7.21 (1.15). The majority of studies (*n* = 18) were rated as high quality (score ≥ 8), suggesting that the overall methodological quality of the included studies was acceptable (Table 3).

**TABLE 3:** Quality assessment of included studies.

Author (year)	Q1	Q2	Q3	Q4	Q5	Q6	Q7	Q8	Q9	Q10	Q11	Score
Zeng et al., 2024 [28]	1	1	1	1	0	1	0	1	0	1	0	7
Dong et al., 2025 [16]	1	1	1	1	0	1	1	1	0	1	0	8
Gao, 2024 [29]	1	0	1	0	0	0	1	0	1	1	0	5
Gong et al., 2022 [30]	1	1	1	0	1	1	1	0	1	1	0	8
Guo et al., 2022 [31]	1	1	1	0	0	0	1	1	1	1	0	7
Han et al., 2024 [32]	1	1	1	0	0	0	1	1	1	1	0	7
He et al., 2023 [20]	1	1	1	0	0	1	1	1	0	1	0	7
Hou et al., 2021 [33]	1	1	1	1	1	0	1	1	0	1	0	8
Hu et al., 2024 [21]	1	1	1	0	0	1	1	1	0	1	0	7
Ji et al., 2025 [15]	1	1	1	0	0	1	1	0	0	1	0	6
Jia et al., 2024 [34]	1	0	1	1	1	1	1	0	1	1	0	8
Li et al., 2024a [35]	1	0	1	1	1	0	1	1	1	1	0	8
Liang et al., 2022 [36]	1	1	1	1	0	0	1	0	1	1	0	7
Shi et al., 2021 [37]	1	0	1	1	0	1	1	1	1	1	0	8
Su et al., 2023 [38]	1	1	1	1	0	1	1	0	1	1	0	8
Luo, 2024 [39]	1	0	1	1	1	1	1	0	1	1	0	8
Su, 2021 [40]	1	1	1	1	0	1	1	0	0	1	0	7
Tian et al., 2021a [19]	1	1	0	0	0	1	1	0	0	1	0	5
Tian et al., 2021b [41]	1	1	1	1	1	1	1	0	0	1	0	8
Yue et al., 2023 [42]	1	1	1	1	0	0	1	1	1	0	0	7
Zheng et al., 2021 [43]	1	1	1	1	1	1	1	0	0	1	0	8
Mo et al., 2024 [17]	1	1	1	1	1	0	0	1	1	1	0	8
Sun et al., 2025 [18]	1	1	1	1	1	1	1	0	0	1	0	8
Wang et al., 2024 [14]	1	0	1	1	0	1	1	1	1	1	0	8
Yang et al., 2025 [44]	1	1	1	1	1	1	1	1	0	1	0	9
Li et al., 2024b [45]	1	0	1	1	1	0	1	1	1	1	0	8
Yu et al., 2024 [46]	1	1	1	1	0	1	1	0	1	1	0	8
Li, 2023 [47]	1	1	1	0	0	1	1	1	1	1	0	8
Li et al., 2022 [48]	1	1	1	0	0	1	0	0	0	0	0	4
Chen et al., 2026 [49]	1	0	1	0	0	1	1	1	1	1	0	7
Xu et al., 2023 [50]	1	1	1	0	0	1	0	1	0	1	0	6
Li et al., 2024c [51]	1	1	1	0	0	1	1	1	1	1	0	8
Dai et al., 2023 [52]	1	1	1	0	0	0	0	1	0	1	0	5
Xie et al., 2021 [53]	1	1	1	0	0	0	1	0	1	1	0	6

*Note:* Yes‐1, No/Unclear‐0. Q1: Source of research subjects; Q2: Eligibility criteria; Q3: Time period of the study population; Q4: Continuity of the study population; Q5: Other circumstances of the research subjects; Q6: Reassessment; Q7: Rationale for the exclusion analysis; Q8: Confounding factor control measures; Q9: Lost data processing; Q10: Response and data collection integrity; and Q11: Follow‐up.

### 3.3. Results of the Meta‐Analysis

#### 3.3.1. Current Status of Medical Narrative Competence Among Clinical Nurses in China

Thirty‐four included studies reported the mean total score of medical narrative competence among Chinese clinical nurses. Substantial heterogeneity was observed (*I* ^2^ = 98.5%, *p* < 0.001); therefore, a random‐effects model was used. The pooled mean total score was 147.38 (95% CI: 145.17–149.60), indicating that medical narrative competence was at the lower end of the moderate level (Figure 2).

**FIGURE 2:**
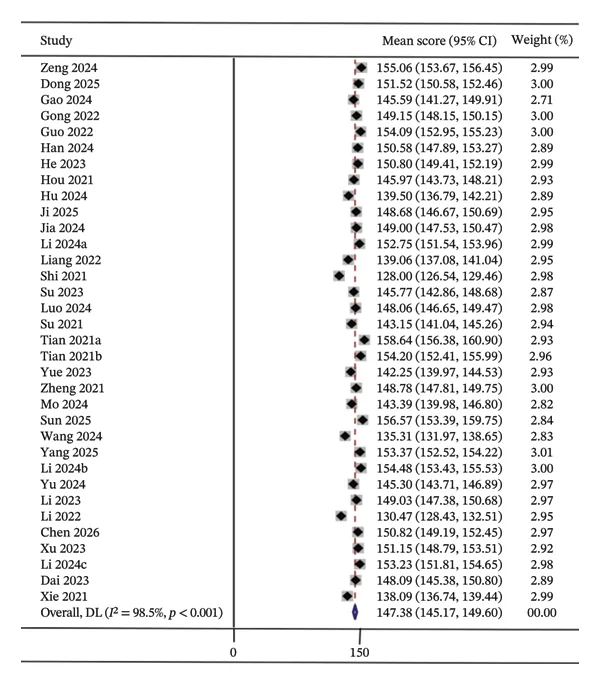
Forest plot of the pooled mean total score of medical narrative competence among Chinese clinical nurses. Note: *p* = *p* value; CI = confidence interval.

#### 3.3.2. Subgroup Analysis

Subgroup analyses were stratified by survey start year, clinical department, and geographic region. All subgroup analyses were performed using a random‐effects model. Subgroup analysis stratified by the starting year of the survey included 32 studies with a total of 21,113 nurses. The studies by Tian et al. [19] and Mo et al. [17] did not report the survey time, and included 220 and 122 nurses, respectively, and were therefore excluded from this subgroup analysis. The pooled mean total scores were 135.56 points (95% CI: 120.71–150.40) for studies with data collection beginning in 2019, 146.02 points (95% CI: 143.35–148.70) for studies with data collection beginning from 2020 to 2022, and 151.69 points (95% CI: 150.07–153.31) for studies with data collection beginning from 2023 to 2025. The point estimates were numerically higher in studies with later survey start years (Supporting Appendix 1, Section S2). Data for the subgroup analysis stratified by department were derived from departmental grouping results reported in the univariate analyses of included primary studies. To ensure data comparability among studies within each subgroup, studies were included in the corresponding department subgroup only if the same department grouping was reported in their univariate analysis and extractable data for pooled analysis were available. Among the 34 included studies, 15 reported consistent department grouping. Ultimately, 23 combinable department datasets were obtained: 3 for outpatient departments; 8 for general medical and surgical wards; 6 for obstetrics, gynecology, and pediatrics; and 6 for emergency departments and ICUs. The remaining 19 studies adopted department grouping schemes that were inconsistent with those of the 15 studies above and could not be assigned to the same department subgroups; therefore, they were excluded from this subgroup analysis. The pooled results showed that the pooled medical narrative competence score of nurses in obstetrics, gynecology, and pediatrics was 149.44 (95% CI: 143.80–155.09), that of nurses in general medical and surgical wards was 149.08 (95% CI: 146.13–152.03), that of nurses in emergency departments and ICUs was 149.07 (95% CI: 145.34–152.80), and that of outpatient nurses was 137.80 (95% CI: 122.79–152.80). According to the point estimates of pooled scores, nurses working in obstetrics, gynecology, and pediatrics had the highest score; scores of nurses in general medical and surgical wards were close to those in emergency departments and ICUs, while outpatient nurses had the lowest score (Supporting Appendix 1, Section S3). The geographic subgroup analysis included 33 studies involving 19,624 nurses, as the study by Li et al. [45] (*n* = 1831) did not report the province in which it was conducted. The pooled mean total scores were 147.72 (95% CI: 142.59–152.85) in northern China and 146.83 (95% CI: 144.64–149.01) in southern China (Table 4; Supporting Appendix 1, Section S4).

**TABLE 4:** Subgroup analysis of the current status of medical narrative competence among clinical nurses in China.

Subgroup	No. of contributing studies	Sample size	Heterogeneity		Meta‐analysis results	
			*I* ^2^ (%)	*p*	Pooled mean total score (95% CI)	*p*
*Survey start year*						
2019	2	802	99.3	< 0.001	135.56 (120.71, 150.40)	< 0.001
2020 to 2022	20	13,326	98.3	< 0.001	146.02 (143.35, 148.70)	< 0.001
2023 to 2025	10	6985	91.8	< 0.001	151.69 (150.07, 153.31)	< 0.001

*Department*						
Outpatient department	3	97	93.6	< 0.001	137.80 (122.79, 152.80)	< 0.001
General Internal Medicine and General Surgery	8	2916	94.0	< 0.001	149.08 (146.13, 152.03)	< 0.001
Obstetrics and Gynecology and Pediatrics	6	399	91.6	< 0.001	149.44 (143.80, 155.09)	< 0.001
Emergency Department and Intensive Care Unit	6	661	76.6	< 0.001	149.07 (145.34, 152.80)	< 0.001

*Region*						
North	13	6337	99.1	< 0.001	147.72 (142.59, 152.85)	< 0.001
South	20	13,287	97.5	< 0.001	146.83 (144.64, 149.01)	< 0.001

*Note:* The subgroup stratified by survey time was grouped according to the starting survey time reported in each primary study. The department subgroup was based on internal department stratification within each primary study. A single study could contribute data to multiple department subgroups; therefore, the sum of studies across all department subgroups may exceed the total number of studies included in the department subgroup analysis.

#### 3.3.3. Analysis of Influencing Factors on Medical Narrative Competence Among Clinical Nurses in China

Influencing factors reported in at least five studies were included in the pooled analysis, yielding 17 factors. Meta‐analysis results showed that clinical nurses aged > 35 years (MD = 4.00, 95% CI: 0.78–7.22), with working experience ≥ 10 years (MD = 4.77, 95% CI: 3.22–6.31), holding senior professional titles (MD = 7.26, 95% CI: 3.34–11.19), in head nurse or higher positions (MD = 4.85, 95% CI: 2.32–7.39), male nurses (MD = 3.18, 95% CI: 0.08–6.27), with monthly income ≥ 10,000 CNY (MD = 4.80, 95% CI: 0.66–8.93), being very familiar with NM (MD = 20.25, 95% CI: 16.95–23.54), and having received narrative nursing training experience (MD = 9.49, 95% CI: 5.68–13.30) exhibited higher medical narrative competence. By contrast, clinical nurses with moderate colleague support (MD = −15.35, 95% CI: −20.43 to −10.26) and dissatisfaction with nursing work (MD = −18.10, 95% CI: −21.16 to −15.03) had lower medical narrative competence. All these differences were statistically significant (*p* < 0.05). No significant associations were found for hospital grade, educational background, employment type, status as an only child, marital status, family background, or childbearing status. Detailed results are presented in Table 5, and factor‐specific forest plots are provided in Supporting Appendix 1, Section S5.

**TABLE 5:** Meta‐analysis of influencing factors of medical narrative competence among Chinese clinical nurses (between‐group comparison).

Influencing factors	Experimental group	Control group	Number of studies	Heterogeneity		Model	Meta‐analysis results	
				*I* ^2^ (%)	*p*		MD (95% CI)	*p*
Age (years)	> 35	≤ 35	14	91	< 0.001	Random	4.00 (0.78, 7.22)	0.01
Working years	≥ 10	≤ 5	27	69	< 0.001	Random	4.77 (3.22, 6.31)	**< 0.001**
Professional title	Senior	Junior	24	90	< 0.001	Random	7.26 (3.34, 11.19)	**< 0.001**
Degree	College and below	Bachelor and above	24	84	< 0.001	Random	−0.57 (−2.69, 1.56)	0.60
Post	Head nurse and above	Frontline nurses	12	65	< 0.001	Random	4.85 (2.32, 7.39)	**< 0.001**
Status as an only child	Yes	No	6	0	0.42	Fixed	−1.67 (−3.55, 0.22)	**0.08**
Marital status	Other	Married	25	99	< 0.001	Random	−4.61 (−10.73, 1.51)	0.14
Colleague concern	Moderate concern	High concern	10	89	< 0.001	Random	−15.35 (−20.43, −10.26)	**< 0.001**
Gender	Male	Female	20	69	< 0.001	Random	3.18 (0.08, 6.27)	**0.04**
Familiar with narrative medicine	Very Familiar	No Exposure	19	69	< 0.001	Random	20.25 (16.95, 23.54)	**< 0.001**
Fertility status	Nulliparous	Parous	11	99	< 0.001	Random	−3.51 (−11.29, 4.27)	0.38
Grade of medical institution	Grade III hospital	Grade II hospital	5	56	0.06	Random	1.95 (−0.42, 4.33)	**0.11**
Employment method	Civil servant	Contract system	8	64	0.007	Random	0.19 (−2.03, 2.41)	0.87
Income (RMB/month)	≥ 10,000	≤ 5000	8	68	0.003	Random	4.80 (0.66, 8.93)	**0.02**
Narrative Nursing Training (Knowledge) Experience	Yes	No	14	96	< 0.001	Random	9.49 (5.68, 13.30)	**< 0.001**
Nursing Job Satisfaction	Dissatisfied	Satisfied	6	0	0.75	Fixed	−18.10 (−21.16, −15.03)	**< 0.001**
Family background	Urban	Rural	5	90	< 0.001	Random	−2.08 (−7.06, 2.90)	0.41

*Note:* The bold values indicate statistically significant results (*p* < 0.05).

Abbreviation: NR, not reported.

#### 3.3.4. Sensitivity Analysis

Sensitivity analyses for the pooled mean score were performed using the leave‐one‐out method. As shown in Figure 3, the pooled mean total score remained stable after omitting each individual study, indicating the robustness of the overall estimate. Additional sensitivity analyses comparing fixed‐effects and random‐effects models were conducted for the following variables: years of clinical experience, professional rank, monthly income, gender, coworker support, age, and other influencing factors. No substantial changes were observed in the MD values or their 95% confidence intervals (95% CI) between the two models, suggesting that the pooled results were robust to the choice of statistical model (Table 6).

**FIGURE 3:**
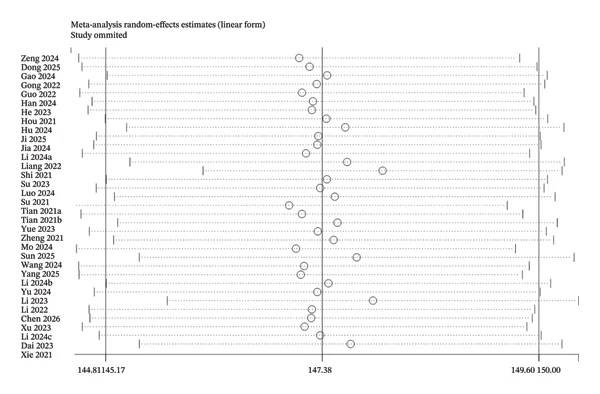
Sensitivity analysis of the current status of medical narrative competence among Chinese clinical nurses.

**TABLE 6:** Sensitivity analysis of influencing factors of medical narrative competence among Chinese clinical nurses.

Influencing factors	Fixed‐effects model (MD, 95% CI)	Random‐effects model (MD, 95% CI)
Working years	4.69 (3.89, 5.48)	4.77 (3.22, 6.31)
Professional title	6.68 (5.50, 7.85)	7.26 (3.34, 11.19)
Post	4.04 (2.77, 5.32)	4.85 (2.32, 7.39)
Gender	3.85 (2.30, 5.41)	3.18 (0.08, 6.27)
Colleague concern	−14.55 (−16.11, −12.98)	−15.35 (−20.43, −10.26)
Age	5.14 (4.21, 6.06)	4.00 (0.78, 7.22)
Familiar with narrative medicine	20.26 (18.55, 21.97)	20.25 (16.95, 23.54)
Income (RMB/month)	4.71 (2.44, 6.97)	4.80 (0.66, 8.93)
Narrative Nursing Training (Knowledge) Experience	11.33 (10.59, 12.08)	9.49 (5.68, 13.30)
Nursing Job Satisfaction	−18.10 (−21.16, −15.03)	−18.10 (−21.16, −15.03)

#### 3.3.5. Publication Bias

Publication bias in the meta‐analysis of the overall mean NCS score among Chinese clinical nurses was assessed using funnel plots, Begg’s test, and Egger’s test. Visual inspection of the funnel plot revealed obvious asymmetry, suggesting potential publication bias in this meta‐analysis (Figure 4). Formal quantitative assessments were subsequently conducted using Begg’s test and Egger’s test. Begg’s test yielded *Z* = −2.56 (*p* = 0.01), indicating significant publication bias; Egger’s test yielded *t* = −1.97 (*p* = 0.06), showing marginal statistical significance and suggesting a moderate risk of publication bias. The trim‐and‐fill method was applied to assess and adjust for publication bias. The pooled mean total score remained largely unchanged after adjustment: under the random‐effects model, the pooled mean total score was 147.38 (95% CI: 145.17–149.60, *p* < 0.001) before correction and 145.63 (95% CI: 143.26–148.00, *p* < 0.001) after correction. This finding suggests that publication bias had no material impact on the overall pooled estimate, supporting the robustness of our conclusions. Publication bias was assessed for 11 factor‐specific meta‐analyses, each including at least 10 studies. Egger’s test indicated funnel plot asymmetry for marital status (*t* = 2.53, *p* = 0.019) and childbearing status (*t* = 3.39, *p* = 0.008), suggesting possible publication bias. No statistical evidence of asymmetry was detected for the remaining factors. The factor‐specific funnel plots and the corresponding Begg’s and Egger’s test outputs are provided in Supporting Appendix 1, Section S6.

**FIGURE 4:**
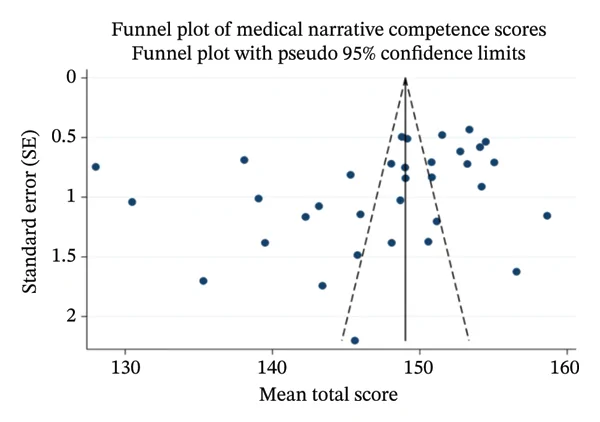
Funnel plot of medical narrative competence among Chinese clinical nurses.

## 4. Discussion

This meta‐analysis quantitatively synthesized evidence on the overall level and influencing factors of medical narrative competence among Chinese clinical nurses. Systematic searches across multiple electronic databases identified 34 eligible studies involving 21,455 clinical nurses. The results showed that the pooled mean score of medical narrative competence among clinical nurses in China was 147.38 (95% CI: 145.17–149.60), which falls at the lower end of the moderate range. There were certain differences across survey starting years, geographical regions, and clinical departments. Among the 17 associated factors included in the analysis, 10 showed statistically significant pooled between‐group differences. Nurses aged > 35 years, with working experience ≥ 10 years, holding senior professional titles, working as head nurses or in higher positions, male nurses, those with a monthly income ≥ 10,000 CNY, those very familiar with NM, those who had received narrative nursing (knowledge) training, nurses with high colleague support, and nurses satisfied with nursing work achieved higher scores on medical narrative competence. No statistically significant between‐group differences were observed for hospital grade, educational level, employment type, only‐child status, marital status, fertility status, and family background. The above findings provide a basis for identifying characteristics associated with clinical nurses’ medical narrative competence and developing targeted training and management strategies. However, since all included studies were cross‐sectional, these results reflect only statistical associations between groups, and causality cannot be inferred from them.

Subgroup analysis stratified by survey starting year showed that the pooled mean scores of medical narrative competence among clinical nurses in the 2019, 2020–2022, and 2023–2025 groups were 135.56, 146.02, and 151.69, respectively. Studies conducted in later periods presented numerically higher scores. However, these between‐study differences may be influenced by sample composition and research settings, so a continuous improvement in nurses’ medical narrative competence over time cannot be inferred. Since its introduction to China in 2011 [3], NM has gradually expanded from theoretical introduction to educational training and clinical application [54]. This developmental background may partially explain the above differences, but its effect requires further verification. At the practical level, nursing managers may consider integrating NM training into the standardized training for newly recruited nurses and the continuing education for practicing nurses, and designing stratified curricula based on clinical experience and actual training needs.

Pooled medical narrative competence scores differed across geographic regions, with nurses in northern China showing a slightly higher mean score (147.72) than those in southern China (146.83). Nevertheless, interpretation of this regional difference must account for the statistical properties of the two subgroups. The southern subgroup had a larger total sample size (*n* = 13,287) and a narrower 95% confidence interval (95% CI: 144.64–149.01), reflecting greater precision of the pooled estimate. In contrast, the northern subgroup had a smaller sample (*n* = 6337) and very high between‐study heterogeneity (*I* ^2^ = 99.1%), with a wide 95% CI (95% CI: 142.59–152.85), suggesting that the pooled result may be unduly influenced by outlying studies. Accordingly, the observed regional difference may reflect disparities in sample characteristics and statistical precision between the two subgroups, rather than a substantive gap in medical narrative competence between clinical nurses in northern versus southern China. Clinically, most southern studies were conducted in tertiary Grade‐A hospitals located in densely populated provinces such as Guangdong, Hunan, and Sichuan. High patient volumes and demanding nursing workloads in these settings may limit the time available for narrative‐based patient care [44, 55]. Overall, this meta‐analysis characterizes regional patterns in medical narrative competence among Chinese clinical nurses. Given the imbalance across subgroups in statistical power, departmental distribution, and sample characteristics, definitive conclusions cannot be drawn regarding higher medical narrative competence in northern versus southern nurses. Future multicenter comparative studies with large samples across northern and southern China are warranted. These studies should account for confounding variables such as department type, hospital level, and years of clinical experience to inform the development of tailored NM training programs.

Subgroup analysis stratified by clinical department showed that the pooled medical narrative competence scores were similar among nurses in obstetrics/gynecology and pediatrics, general medical and surgical wards, and emergency departments/ICUs, with values of 149.44, 149.08 and 149.07, respectively. The outpatient group had a relatively lower pooled score of 137.80. The comparable scores observed in the first three groups may stem from shared features of their clinical care settings and similar demands for narrative communication. However, practical opportunities for narrative communication still vary across department contexts. In contrast, outpatient care mainly consists of triage, patient guidance, and health education. The short nurse–patient contact time, fast workflow, and, to some extent, fragmented settings restrict the implementation of in‐depth narrative communication [56]. Nevertheless, this explanation requires further verification, and the pooled subgroup scores alone cannot confirm an independent association between department affiliation and medical narrative competence. Notably, the outpatient subgroup included only three studies with a total of 97 nurses, a wide 95% CI (122.79–152.80), and high within‐subgroup heterogeneity (*I* ^2^ = 93.6%), which limited the precision and stability of the pooled estimate. Therefore, it is inappropriate to conclude that outpatient nurses have lower medical narrative competence than nurses in other departments. Furthermore, we should not infer a widespread deficiency in medical narrative competence among Chinese clinical nurses merely because the pooled mean score of each subgroup did not reach the high‐level range. Future multicenter, large‐sample studies are warranted to explore further the association between department type and medical narrative competence after adjusting for potential confounders such as working years, job responsibilities and clinical work environment. At the practical level, nursing managers may combine competence assessment results with training needs. Based on universal foundational training covering listening, empathy, and reflection, tailored training programs can be developed for different departmental scenarios. Given the limited nurse–patient contact time and fast diagnostic pace in outpatient settings, short‐duration narrative communication training and scenario simulations can be implemented to help nurses complete key narrative interactions within the limited consultation time, thereby improving the targeting and the clinical suitability of training.

This meta‐analysis identified multiple key influencing factors of medical narrative competence among Chinese clinical nurses, categorized into two broad dimensions: individual characteristics and occupational correlates. Personal characteristics mainly included age, years of experience, professional title, position, and gender. Work‐related factors mainly included monthly income, familiarity with NM, experience with narrative nursing (knowledge) training, colleague support, and satisfaction with nursing work.

Age and years of clinical practice were significantly associated with medical narrative competence. Nurses aged > 35 years and those with working experience ≥ 10 years obtained higher medical narrative competence scores than nurses aged ≤ 35 years and those with working experience ≤ 5 years, respectively, which was broadly consistent with findings from previous studies [45]. These associations may be attributable to accumulated communication experience and enhanced emotional regulation developed through long‐term clinical practice. Older age is associated with greater emotional stability, which may enable nurses to maintain humanistic care in high‐stress clinical settings [57]. In addition, greater clinical experience may provide more opportunities to engage with patients’ illness narratives and complex communication situations, potentially contributing to the development of medical narrative competence. This observation is consistent with the conceptualization of medical narrative competence as a practical, experience‐based competence [16, 29]. Healthcare institutions may consider developing tiered training programs tailored to nurses’ levels of clinical experience (rather than age). Less experienced nurses may benefit from foundational training in NM, simulation‐based practice, and case‐based supervision. More experienced nurses may serve as mentors in narrative practice, facilitating peer learning and supporting the development of medical narrative competence across the team.

Nurses with senior professional titles and those in head nurse or higher positions obtained higher medical narrative competence scores than nurses with junior professional titles and frontline clinical nurses, respectively. Previous studies suggest that nurses with senior professional ranks and administrative positions may have greater access to continuing education and NM training, engage in more complex communication tasks, and report stronger professional identity and self‐efficacy [39, 44, 58], These factors may provide more opportunities for narrative practice and reflective learning. Furthermore, nurse managers may play a key role in disseminating NM concepts and fostering positive team communication climates [17]. They may also demonstrate greater medical narrative competence compared with frontline clinical nurses. In light of these findings, healthcare institutions could consider incorporating medical narrative competence into performance evaluations and promotion criteria for professional rank and administrative positions. Targeted NM education should be strengthened for nurses holding junior professional ranks and those serving in frontline clinical roles. In addition, advanced training in narrative leadership should be provided to nurse managers to support their role in advancing NM within clinical teams.

This meta‐analysis pooled 20 studies with a total sample size of 11,579. The results showed that male nurses had higher medical narrative competence scores than female nurses (MD = 3.18, 95% CI: 0.08–6.27). This finding was contrary to the conclusion of Li et al. [22], who, based on two studies with a sample size of 643, reported that female nurses had higher medical narrative competence scores.

It is commonly assumed that women possess stronger empathy and communication skills, putting them at an advantage in medical narrative competence. The opposite finding in the present study may be related to the real‐world characteristics of the included samples. The substantial difference in sample sizes between the two meta‐analyses may also be an important contributor to the divergent results. In the pooled sample of the present study, male nurses were mostly concentrated in emergency departments, ICUs, and operating rooms, all of which are critical care settings. These posts involve more frequent clinical conflicts and a wider range of nurse–patient communication scenarios, and long‐term high‐intensity clinical communication practice may cultivate their narrative communication abilities. However, the lower limit of the 95% confidence interval was close to zero, and substantial heterogeneity was observed (*I* ^2^ = 69%). Therefore, this finding cannot be interpreted as an inherent competence advantage among male nurses. Gender disparities in medical narrative competence may be attributable to multiple factors, including departmental distribution, job responsibilities, clinical experience, and training opportunities, which require further validation in studies with rigorous control of confounding variables. Therefore, nursing managers should develop targeted training programs based on the actual competence and training needs of clinical nurses, rather than using gender as the sole evaluation and training criterion.

Clinical nurses with a monthly income of ≥ 10,000 yuan had higher medical narrative competence scores than those with a monthly income of ≤ 5000 yuan. Previous research suggests that higher earnings may reduce psychological distress and occupational burnout related to financial stress, thereby contributing to greater perceived job security and work satisfaction [37]. These factors may facilitate engagement in patient‐centered communication and narrative practice. However, some studies did not find this association [43], possibly due to single‐center sampling and regional variations in salary structures. Meanwhile, higher income may accompany senior professional titles, longer working years, and higher job ranks. Thus, score differences across income subgroups may partly reflect disparities in clinical experience, job responsibilities, and career development opportunities. Since this meta‐analysis pooled data from univariate group comparisons in original studies without simultaneous adjustment for the above potential confounding variables, an independent association between monthly income and medical narrative competence cannot be confirmed. Likewise, we cannot infer that higher income improves medical narrative competence.

Familiarity with NM and experience of narrative nursing (knowledge) training were both significantly associated with medical narrative competence scores. Nurses who were very familiar with NM and those who had received relevant training achieved higher scores than nurses who had never been exposed to NM and those without relevant training, respectively. These results are consistent with previous findings [14, 20, 38]. Greater familiarity with NM may facilitate the application of active listening, empathy, reflective practice, and other narrative care principles in clinical settings [59]. Structured narrative nursing training may enhance medical narrative competence by imparting both theoretical knowledge and practical skills [30]. The scoping review conducted by Palla et al. [12] reported potential benefits of NM education for empathy, reflection, and therapeutic relationships. Healthcare institutions may consider integrating NM education into continuing professional development programs and offering training that covers both theoretical foundations and applied clinical skills for nurses.

This meta‐analysis found that nurses with high perceived colleague support and satisfaction with nursing work achieved significantly higher medical narrative competence scores. Based on organizational support theory, adequate colleague support can not only buffer work stress but also promote the sharing of professional experience among nurses, create a safe environment for reflective practice, and thereby facilitate the cultivation of narrative competence [33, 60]. Work satisfaction may be linked to professional identity and work engagement [35]. A qualitative study of an NM program conducted by Small et al. [61] indicated that participants, including nurses, perceived that the program helped reduce feelings of isolation at work, enhanced mutual understanding among colleagues, and provided support for self‐care. These participatory experiences complement our discussion of team support, yet they do not directly verify the associations among colleague support, job satisfaction, and medical narrative competence scores. Therefore, nursing managers may create conditions for narrative practice by improving peer support mechanisms, optimizing work environments and staffing allocation, and expanding career development pathways. The effects of such measures on nurses’ medical narrative competence remain to be further evaluated.

In summary, this meta‐analysis identified multiple factors associated with medical narrative competence among Chinese clinical nurses. These relationships were integrated into the conceptual framework shown in Figure 5. Nursing managers can deliver targeted training and improve organizational support and clinical work environments based on nurses’ actual competence and training needs. The findings can provide references for nursing management practice in China and generate testable hypotheses for relevant research in other countries and regions.

**FIGURE 5:**
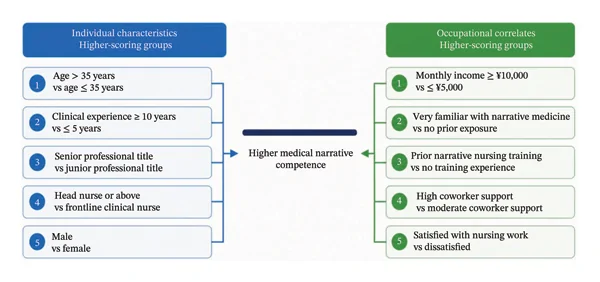
Influencing factors of medical narrative competence among Chinese clinical nurses.

## 5. Limitations

This study has several limitations. First, all included studies were cross‐sectional, so the results reflect only statistical associations and cannot be used to infer causal relationships. Second, this meta‐analysis included only samples of practicing clinical nurses in China, and no cross‐national quantitative comparison was performed. Differences in nursing education, professional roles, and clinical culture across healthcare systems may limit the generalizability of the findings. Third, substantial variability existed across the included studies in geographic distribution, clinical department composition, and sample characteristics. Subgroup analyses failed to fully account for the high between‐study heterogeneity, which may limit the precision of our pooled estimates. Future multinational, multicenter longitudinal or interventional studies should be conducted. Meanwhile, greater efforts are needed to standardize measurements and complete cross‐cultural validation of assessment tools, to improve comparability across studies, and to further examine the cross‐cultural applicability of these associations and conclusions.

## 6. Conclusion

This systematic review and meta‐analysis revealed that the medical narrative competence of clinical nurses in China remains at the lower end of the moderate level. Group comparisons revealed that nurses aged > 35 years, with working experience ≥ 10 years, holding senior professional titles, working as head nurses or in higher positions, male nurses, those with monthly income ≥ 10,000 yuan, those very familiar with NM, those with narrative nursing (knowledge) training experience, nurses reporting high colleague support, and nurses satisfied with nursing work had higher medical narrative competence scores than their corresponding reference groups. The pooled correlates offer empirical references for formulating nursing management strategies, supporting capacity‐building of medical narrative competence among clinical nurses, and advancing overall nursing care quality. They may also serve as suggestive evidence for future research and clinical decision‐making. In routine clinical practice, nursing administrators may use these observed correlates to screen nurses at potential risk of suboptimal narrative competence and implement tailored interventions and educational training aimed at improving narrative‐related performance and patient care outcomes. Nevertheless, how these correlates may relate to individual subdimensions of medical narrative competence remains poorly understood. Further fine‐grained investigations are needed to explore potential differentiated associations across different subdimensions. Drawing upon this set of observed correlates, more person‐tailored intervention approaches could be explored in subsequent work.

## Author Contributions

Yuqin Li and Rong Hu contributed equally to this article as co‐first authors, with Yuqin Li listed as the first co‐author and Rong Hu as the second co‐author.

## Funding

No funds were received.

## Conflicts of Interest

The authors declare no conflicts of interest.

## Supporting Information

Additional supporting information can be found online in the Supporting Information section.

## Supporting information


**Supporting Information 1** One supporting file (Supporting Appendix 1) accompanies this manuscript. It contains six sections (S1–S6), described below and cited at the relevant points in the main text. Section S1. Full search strategies and retrieval counts for the 11 databases (3079 records identified; last search: 30 August 2026). Section S2. Forest plots of pooled NCS total scores by survey start year (2019, 2020–2022, and 2023–2025). Section S3. Forest plots of pooled NCS total scores by clinical department (outpatient; general medical/surgical; obstetrics/gynecology/pediatrics; and emergency/ICU). Section S4. Forest plots of pooled NCS total scores by geographic region (northern and southern China). Section S5. Forest plots of pooled mean differences in NCS total scores for 17 influencing factors. Section S6. Funnel plots and Begg’s and Egger’s test outputs for 11 factor‐specific meta‐analyses (at least 10 studies each).

**Supporting Information 2** PRISMA‐checklist.

## Data Availability

The data that support the findings of this study are available from the corresponding author upon reasonable request.
