# Supplementary material for: Influencing Factors of Medical Narrative Competence Among Clinical Nurses in China: A Systematic Review and Meta‐Analysis
Source: J Nurs Manag. 2026 Nov 3;2026:5040520. doi: 10.1155/jonm/5040520 (PMC13728992; doi:10.1155/jonm/5040520)
Supplement: Supplementary file 2 [file JONM-2026-5040520-s002.docx]

**Contents of supplementary appendix**

| S1 | Search strategy and results | Page1-5 |
| --- | --- | --- |
| S2 | Subgroup analysis based on Year | Page6-7 |
| S3 | Subgroup analysis based on Department | Page8-9 |
| S4 | Subgroup analysis based on Region | Page9 |
| S5 | Forest Plot of Influencing Factors of Medical Narrative Competence Among Clinical Nurses in China | Page10-14 |
| S6 | Funnel Plot of Influencing Factors Analysis | Page15-22 |

**S1 Full database search strategies and retrieval counts (11 databases; 3,079 records; last searched on 30 August 2026).**

| **#** | Scopus | **Results** |
| --- | --- | --- |
| #1 | TITLE-ABS-KEY(Nurses OR nurse OR registered nurse OR clinical nurse OR hospital nurse OR staff nurse OR nurse clinician OR professional nurse OR nursing personnel OR nursing staff OR hospital nursing staff) | 22,939 |
| #2 | TITLE-ABS-KEY (narrative medicine OR narrative-based medicine OR medical narrative OR medical narrative competence OR clinical narrative competence OR narrative competenc* OR medical narrative ability OR narrative ability OR narrative capacity OR medical narrative literacy OR narrative literacy OR narrative nursing OR narrative care OR narrative writing OR medical narrative writing) | 240 |
| #3 | #1 AND #2 | 11 |

| **#** | Pubmed | **Results** |
| --- | --- | --- |
| #1 | (("Nurses"[Mesh])) OR ((((((((((nurse[Title/Abstract]) OR (registered nurse[Title/Abstract])) OR (clinical nurse[Title/Abstract])) OR (hospital nurse[Title/Abstract])) OR (staff nurse[Title/Abstract])) OR (nurse clinician[Title/Abstract])) OR (professional nurse[Title/Abstract])) OR (nursing personnel[Title/Abstract])) OR (nursing staff[Title/Abstract])) OR (hospital nursing staff[Title/Abstract])) | 252,334 |
| #2 | ("Narrative Medicine"[Mesh]) OR ((((((((((((((narrative-based medicine[Title/Abstract]) OR (medical narrative[Title/Abstract])) OR (medical narrative competence[Title/Abstract])) OR (clinical narrative competence[Title/Abstract])) OR (narrative competenc*[Title/Abstract])) OR (medical narrative ability[Title/Abstract])) OR (narrative ability[Title/Abstract])) OR (narrative capacity[Title/Abstract])) OR (medical narrative literacy[Title/Abstract])) OR (narrative literacy[Title/Abstract])) OR (narrative nursing[Title/Abstract])) OR (narrative care[Title/Abstract])) OR (narrative writing[Title/Abstract])) OR (medical narrative writing[Title/Abstract])) | 951 |
| #3 | #1 AND #2 | 45 |

| **#** | **Web of science** | **Results** |
| --- | --- | --- |
| #1 | TS=(Nurses OR nurse OR registered nurse OR clinical nurse OR hospital nurse OR staff nurse OR nurse clinician OR professional nurse OR nursing personnel OR nursing staff OR hospital nursing staff) | 274,369 |
| #2 | TS=(narrative medicine OR narrative-based medicine OR medical narrative OR medical narrative competence OR clinical narrative competence OR narrative competenc* OR medical narrative ability OR narrative ability OR narrative capacity OR medical narrative literacy OR narrative literacy OR narrative nursing OR narrative care OR narrative writing OR medical narrative writing) | 57,130 |
| #3 | TS=(risk factors OR potential factor OR associated factor OR influencing factor OR related factor OR relevant factor OR contributing factor OR independent factor OR predictor OR determinant OR correlate* OR associat* OR relationship OR correlation) | 10,384,479 |
| #4 | #1 AND #2 AND #3 | 2,371 |

| **#** | **Cochrane** | **Results** |
| --- | --- | --- |
| #1 | MeSH descriptor: [Nurses] explode all trees | 1,873 |
| #2 | (nurse):ti,ab,kw OR (registered nurse ):ti,ab,kw OR (clinical nurse):ti,ab,kw OR (hospital nurse):ti,ab,kw OR (staff nurse):ti,ab,kw OR (nurse clinician):ti,ab,kw OR (professional nurse):ti,ab,kw OR (nursing personnel):ti,ab,kw OR (nursing staff):ti,ab,kw OR (hospital nursing staff):ti,ab,kw | 33,353 |
| #3 | #1 OR #2 | 33,651 |
| #4 | MeSH descriptor: [Narrative Medicine] explode all trees | 18 |
| #5 | (narrative competenc*):ti,ab,kw OR (medical narrative ability):ti,ab,kw OR (narrative ability):ti,ab,kw OR (narrative capacity):ti,ab,kw OR (medical narrative literacy):ti,ab,kw OR (narrative literacy):ti,ab,kw OR (narrative nursing):ti,ab,kw OR (narrative care):ti,ab,kw OR (narrative writing):ti,ab,kw OR (medical narrative writing):ti,ab,kw OR (narrative medicine):ti,ab,kw OR (narrative-based medicine):ti,ab,kw OR (medical narrative):ti,ab,kw OR (medical narrative competence):ti,ab,kw OR (clinical narrative competence):ti,ab,kw | 1,949 |
| #6 | #4 OR #5 | 1,950 |
| #7 | MeSH descriptor: [Risk Factors] explode all trees | 39,138 |
| #8 | (potential factor):ti,ab,kw OR (associated factor):ti,ab,kw OR (influencing factor):ti,ab,kw OR (related factor):ti,ab,kw OR (relevant factor):ti,ab,kw OR (contributing factor):ti,ab,kw OR (independent factor):ti,ab,kw OR (predictor):ti,ab,kw OR (determinant):ti,ab,kw OR (correlate*):ti,ab,kw OR (associat*):ti,ab,kw OR (relationship):ti,ab,kw OR (correlation):ti,ab,kw | 580,491 |
| #9 | #7 OR #8 | 597,776 |
| #10 | #3 AND #6 AND #9 | 52 |

| **#** | **Embase** | **Results** |
| --- | --- | --- |
| #1 | 'Nurses':ab,ti OR 'nurse':ab,ti OR 'registered nurse ':ab,ti OR 'clinical nurse':ab,ti OR 'hospital nurse':ab,ti OR 'staff nurse':ab,ti OR 'nurse clinician':ab,ti OR 'professional nurse':ab,ti OR 'nursing personnel':ab,ti OR 'nursing staff':ab,ti OR 'hospital nursing staff':ab,ti | 481,754 |
| #2 | 'narrative medicine':ab,ti OR 'narrative-based medicine':ab,ti OR 'medical narrative':ab,ti OR 'medical narrative competence':ab,ti OR 'clinical narrative competence':ab,ti OR 'narrative competenc*':ab,ti OR 'medical narrative ability':ab,ti OR 'narrative ability':ab,ti OR 'narrative capacity':ab,ti OR 'medical narrative literacy':ab,ti OR 'narrative literacy':ab,ti OR 'narrative nursing':ab,ti OR 'narrative care':ab,ti OR 'narrative writing':ab,ti OR 'medical narrative writing':ab,ti | 1,634 |
| #3 | #1 AND #2 | 128 |

| **#** | **CINAHL** | **Results** |
| --- | --- | --- |
| #1 | TI(Nurses OR nurse OR registered nurse OR clinical nurse OR hospital nurse OR staff nurse OR nurse clinician OR professional nurse OR nursing personnel OR nursing staff OR hospital nursing staff) | 222,541 |
| #2 | TI(narrative medicine OR narrative-based medicine OR medical narrative OR medical narrative competence OR clinical narrative competence OR narrative competenc* OR medical narrative ability OR narrative ability OR narrative capacity OR medical narrative literacy OR narrative literacy OR narrative nursing OR narrative care OR narrative writing OR medical narrative writing) | 1,087 |
| #3 | #1 AND #2 | 58 |
| **#** | PsycINFO | **Results** |
| #1 | Title: Nurses OR Title: nurse OR Title: registered nurse OR Title: clinicalnurse OR Title: hospital nurse OR Title: staff nurse OR Title: nurse clinician OR Title:professional nurse OR Title: nursing personnel OR Title: nursing staff OR Title: hospital nursing staff | 30,104 |
| #2 | Title: narrative medicine OR Title: narrative-based medicine ORTitle:medical narrative OR Title: medical narrative competence OR Title: clinical narrativecompetence OR Title: narrative competenc* OR Title: medical narrative ability OR Title:narrative ability OR Title: narrative capacity OR Title: medical narrative literacy ORTitle:narrative literacy OR Title: narrative nursing OR Title: narrative care OR Title: narrative writing OR Title: medical narrative writing | 1,309 |
| #3 | Title: risk factors OR Title: potential factor OR Title: associated factor ORTitle: influencing factor OR Title: related factor ORTitle: relevant factor OR Title: contributingfactor OR Title: independent factor OR Title: predictor OR Title: determinant OR Title: correlate*ORTitle: associat* ORTitle: relationship ORTitle: correlation | 339,554 |
| #4 | #1 AND #2 AND #3 | 2 |

| **#** | **VIP** | **Results** |
| --- | --- | --- |
| #1 | M=(Nurses OR nurse OR registered nurse OR clinical nurse OR hospital nurse OR staff nurse OR nurse clinician OR professional nurse OR nursing personnel OR nursing staff OR hospital nursing staff OR in-service nurse) | 129,057 |
| #2 | M=(narrative medicine OR narrative‑based medicine OR medical narrative OR medical narrative competence OR clinical narrative competence OR narrative competenc* OR medical narrative ability OR narrative ability OR narrative capacity OR medical narrative literacy OR narrative literacy OR narrative nursing OR narrative care OR narrative writing OR medical narrative writing) | 5,746 |
| #3 | M=(risk factors OR potential factor OR associated factor OR influencing factor OR related factor OR relevant factor OR contributing factor OR independent factor OR predictor OR determinant OR correlate* OR associat* OR relationship OR correlation) | 1,046,989 |
| #4 | #1 AND #2 AND #3 | 57 |

| **#** | **SinoMed** | **Results** |
| --- | --- | --- |
| #1 | ("Nurses"[Common fields: Intelligent] OR "nursing personnel"[Common fields: Intelligent] OR "clinical nurses"[Common fields: Intelligent] OR "registered nurses"[Common fields: Intelligent] OR "in-service nurses"[Common fields: Intelligent] OR "clinical nursing staff"[Common fields: Intelligent] OR "nursing workers"[Common fields: Intelligent]) | 279,809 |
| #2 | ("medical narrative competence"[Common fields: Intelligent] OR "clinical narrative competence"[Common fields: Intelligent] OR "narrative competence"[Common fields: Intelligent] OR "medical narrative literacy"[Common fields: Intelligent] OR "narrative literacy"[Common fields: Intelligent] OR "narrative competency"[Common fields: Intelligent] OR "narrative medicine"[Common fields: Intelligent] OR "medical narrative"[Common fields: Intelligent] OR "narrative nursing"[Common fields: Intelligent] OR "narrative care"[Common fields: Intelligent] OR "narrative writing"[Common fields: Intelligent] OR "medical narrative writing"[Common fields: Intelligent] OR "clinical narrative"[Common fields: Intelligent] OR "medical narrative competency"[Common fields: Intelligent] OR "clinical narrative competency"[Common fields: Intelligent]) | 2,773 |
| #3 | ("influencing factors"[Common fields: Intelligent] OR "related factors"[Common fields: Intelligent] OR "associated factors"[Common fields: Intelligent] OR "potential factors"[Common fields: Intelligent] OR "predictive factors"[Common fields: Intelligent] OR "determinants"[Common fields: Intelligent] OR "independent factors"[Common fields: Intelligent] OR "risk factors"[Common fields: Intelligent] OR "correlation"[Common fields: Intelligent] OR "association"[Common fields: Intelligent]) | 1,136,986 |
| #4 | #1 AND #2 AND #3 | 87 |

| **#** | **CNKI** | **Results** |
| --- | --- | --- |
| #1 | Subject=（Nurses OR nurse OR registered nurse OR clinical nurse OR hospital nurse OR staff nurse OR nurse clinician OR professional nurse OR nursing personnel OR nursing staff OR hospital nursing staff OR in-service nurse） | 432,778 |
| #2 | Subject=（narrative medicine OR narrative‑based medicine OR medical narrative OR medical narrative competence OR clinical narrative competence OR narrative competenc* OR medical narrative ability OR narrative ability OR narrative capacity OR medical narrative literacy OR narrative literacy OR narrative nursing OR narrative care OR narrative writing OR medical narrative writing） | 7,330 |
| #3 | Subject=（risk factors OR potential factor OR associated factor OR influencing factor OR related factor OR relevant factor OR contributing factor OR independent factor OR predictor OR determinant OR correlate* OR associat* OR relationship OR correlation） | 2,642,559 |
| #4 | #1 AND #2 AND #3 | 119 |

| **#** | **WANFANG** | **Results** |
| --- | --- | --- |
| #1 | Subject=(Nurses OR nurse OR registered nurse OR clinical nurse OR hospital nurse OR staff nurse OR nurse clinician OR professional nurse OR nursing personnel OR nursing staff OR hospital nursing staff OR in-service nurse) | 671,293 |
| #2 | Subject=(narrative medicine OR narrative‑based medicine OR medical narrative OR medical narrative competence OR clinical narrative competence OR narrative competenc* OR medical narrative ability OR narrative ability OR narrative capacity OR medical narrative literacy OR narrative literacy OR narrative nursing OR narrative care OR narrative writing OR medical narrative writing) | 5,249 |
| #3 | Subject=(risk factors OR potential factor OR associated factor OR influencing factor OR related factor OR relevant factor OR contributing factor OR independent factor OR predictor OR determinant OR correlate* OR associat* OR relationship OR correlation) | 3,281,572 |
| #4 | #1 AND #2 AND #3 | 149 |

**S2：Forest plots of pooled NCS total scores by survey start year**

2019

2020-2022

2023-2025

**S3：Forest plots of pooled NCS total scores by clinical department**

（Outpatient department）

（General Internal Medicine and General Surgery）

（Obstetrics and Gynecology and Pediatrics）

（Emergency Department and Intensive Care Unit）

**S4：Forest plots of pooled NCS total scores by geographic region**

（North）

（South）

**S5：Forest plots of pooled mean differences for factors associated with medical narrative competence**

Working years：

Grade of medical institution：

Professional title：

Degree：

Post：

Status as an only child：

Marital status：

Colleague concern：

Gender：

Familiar with narrative medicine：

Fertility status：

Age(years)：

Employment method：

Income (RMB/month)：

Narrative Nursing Training (Knowledge) Experience：

Nursing Job Satisfaction：

Family background:

**S6：Funnel plots and Begg's and Egger's test outputs for factor-specific meta-analyses**

Working years：

Professional title：

Post：

Age：

Familiar with narrative medicine：

Narrative Nursing Training (Knowledge) Experience：

Colleague concern:

Gender:

Degree:

Marital status:

Fertility status:
